# Artificial Water Point for Livestock Influences Spatial Ecology of a Native Lizard Species

**DOI:** 10.1371/journal.pone.0147433

**Published:** 2016-01-22

**Authors:** Stephan T. Leu, C. Michael Bull

**Affiliations:** School of Biological Sciences, Flinders University, Adelaide, Australia; Curtin University, AUSTRALIA

## Abstract

Pastoralism is a major agricultural activity in drier environments, and can directly and indirectly impact native species in those areas. We investigated how the supply of an artificial watering point to support grazing livestock affected movement and activity patterns of the Australian sleepy lizard (*Tiliqua rugosa*) during a drought year. We observed 23 adult lizards; six had access to a dam, whereas 17 lizards did not. Lizards with access to the dam had larger home ranges, were substantially active on more days (days with >100 steps), and moved more steps per day compared to lizards that did not have access to the dam, both during the early and late period of our observation. Furthermore, while the two groups of lizards had similar body condition early in the season, they differed later in the season. Lizards with dam access retained, whereas lizards without access lost body condition. Local heterogeneity in access to an artificial water resource resulted in spatially dependent behavioural variation among sleepy lizard individuals. This suggests that sleepy lizards have flexible responses to changing climatic conditions, depending on the availability of water. Furthermore, while reducing activity appears a suitable short term strategy, if harsh conditions persist, then access to dams could be of substantial benefit and could support sustained lizard activity and movement and allow maintenance of body condition. Hence, artificial watering points, such as the dams constructed by pastoralists, may provide local higher quality refugia for sleepy lizards and other species during drought conditions.

## Introduction

Human land use, such as agriculture, has far reaching consequences beyond the local level [1]. In 2000 approximately 40 percent of the earth’s ice free terrestrial surface was being used for agriculture [2]. Agricultural activities have been shown to contribute to biodiversity loss, species extinctions and range reductions largely through the replacement of natural ecosystems [3, 4]. In drier rangelands where pastoralism is a major form of agriculture, components of natural ecosystems remain, and certain agricultural activities can have beneficial influence on some native species [3, 5]. Here we investigate how rangeland use, and in particular the supply of an artificial watering point to support grazing of introduced ungulates, affected the spatial ecology and behaviour of a native Australian lizard species.

Artificial watering points, either dams that store the run-off from ephemeral rainfall events, or bores that bring ground water to the surface, are typically used to supply water for grazing livestock in arid and semi-arid areas of Australia. As well as supporting livestock, these watering points allow the persistence of feral species [6], and the spread of invasive species [7]. The invasive cane toad (*Rhinella marina*) for example, has spread into arid Australia using access to man-made open surface water sources, where they have reduced risk of overheating and desiccation [7]. Reptile species are abundant in semi- and arid environments in Australia. Their activity budgets are considered to be mainly driven by ambient temperatures and thermoregulatory behaviour [8–10]. However, reptilian activity is also influenced by hydration and water regulation [10, 11]. For example, most activity in the desert dwelling gila monster (*Heloderma suspectum*) follows rainfall periods [12]. Importantly, several reptile species have been shown to be unable to fully satisfy their water requirements through the food they ingest and through their metabolic cell respiration, but rely, at least temporarily, on access to free standing water or rain [13–15]. This suggests that human built artificial watering points may provide important and more reliable water resources for some native reptiles, particularly in dry areas and during drought conditions. We investigated how an artificial watering point, a human constructed dam affected activity and behaviour in the Australian sleepy lizard (*Tiliqua rugosa*), in a semi-arid habitat in the mid-north region of South Australia, during the drought year 2006.

The sleepy lizard is a large (adult snout–vent length 28 cm) scincid lizard with an estimated lifespan of up to 50 years [16]. Our study area has hot dry summers, and during years with normal rainfall sleepy lizards are most active during spring and early summer (mid-September to mid-December, [8, 17]), and substantially reduce activity after late December [18]. The sleepy lizard is predominantly herbivorous and feeds on annual plants that germinate in early spring and gradually dry out and die off towards summer [19]. This is the time when lizards reduce activity. After prolonged periods without rain, previously inactive sleepy lizards have been observed to respond to rainfall by emerging from their refuges at night, at low ambient temperatures, to opportunistically feed on rehydrated thalli of the terrestrial cyanobacterium *Nostoc commune* and to lick at pools of water on the substrate [20]. This demonstrates both the importance of access to water for this species, and its behavioural flexibility in taking advantage of opportunities for access.

Kerr and Bull [18] found that sleepy lizards were less active and moved less during the drought year 2002, than in a year with more normal precipitation. In the current study we built on this result, and asked how access to a dam affects the behaviour, spatial ecology and body condition of the sleepy lizard during another drought year, 2006. The study site, within rangeland grazed by free-ranging sheep, included one dam, constructed to collect and store run-off from rainfall, and to provide sheep with free standing water. The sheep paddock was much larger than our study site and sheep regularly moved between several other dams. For the resident lizards the dam provided both water, and a surrounding habitat of moister soil that maintained annual plant growth for longer into the summer than the rest of the area. We predicted that lizards that had access to free standing water at the dam would (1) have larger home ranges that included regular visits from their refuges to the dam, (2) be active on more days, and (3) move more per day than lizards that did not have access to these resources; and that this would result in (4) better body condition. We predicted that access to water would play an important role in the spatial ecology of this lizard species, and that dams may provide local refugia for these lizards during extreme drought conditions.

## Material and Methods

### Study site and year

**Fig 1:**
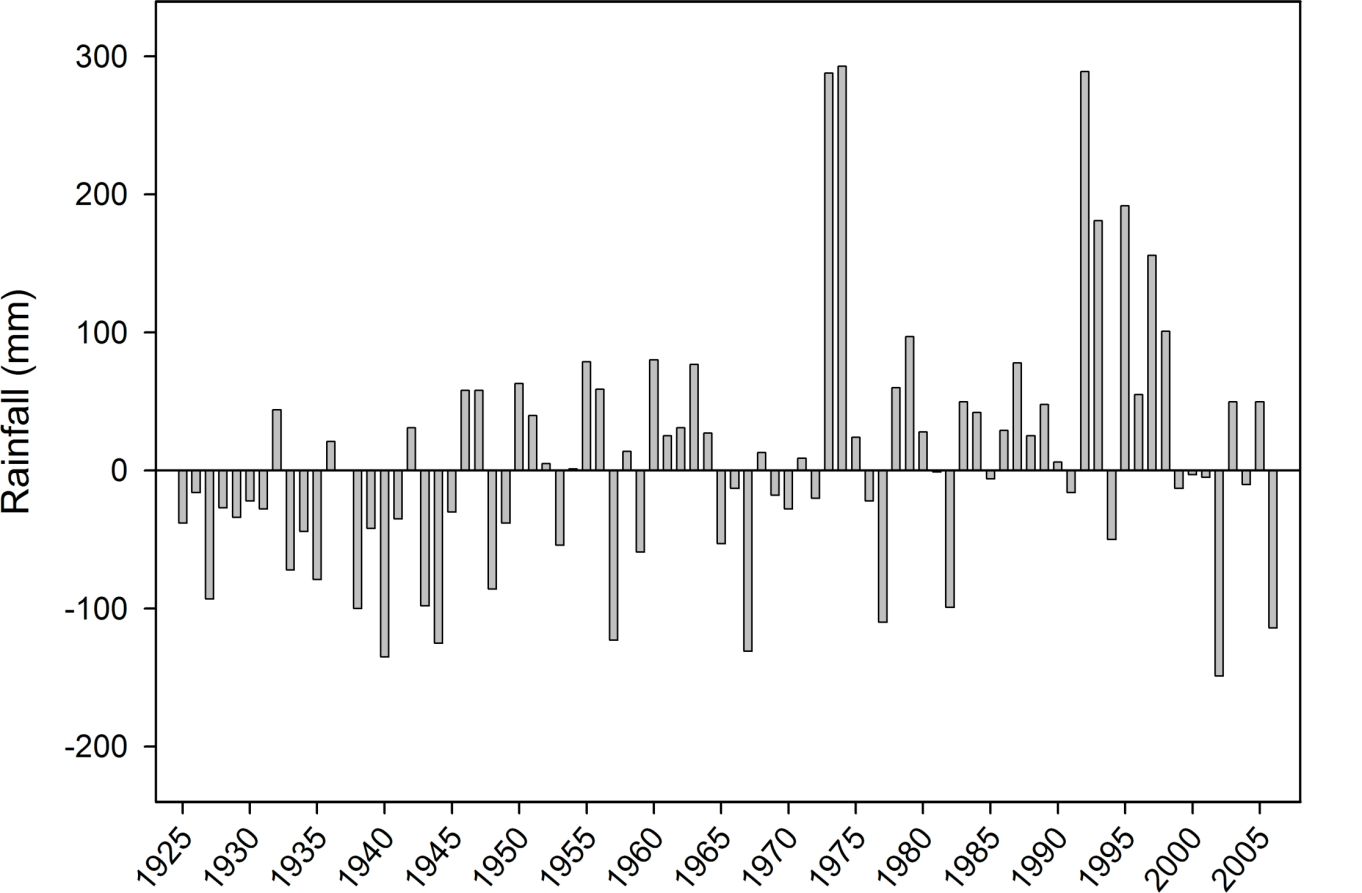
Total annual rainfall (mm), relative to the long-term annual median (230mm, year 1925 to 2005). Rainfall was recorded at Bundey Bore station, approx. 3 km from our field site.

**Fig 2:**
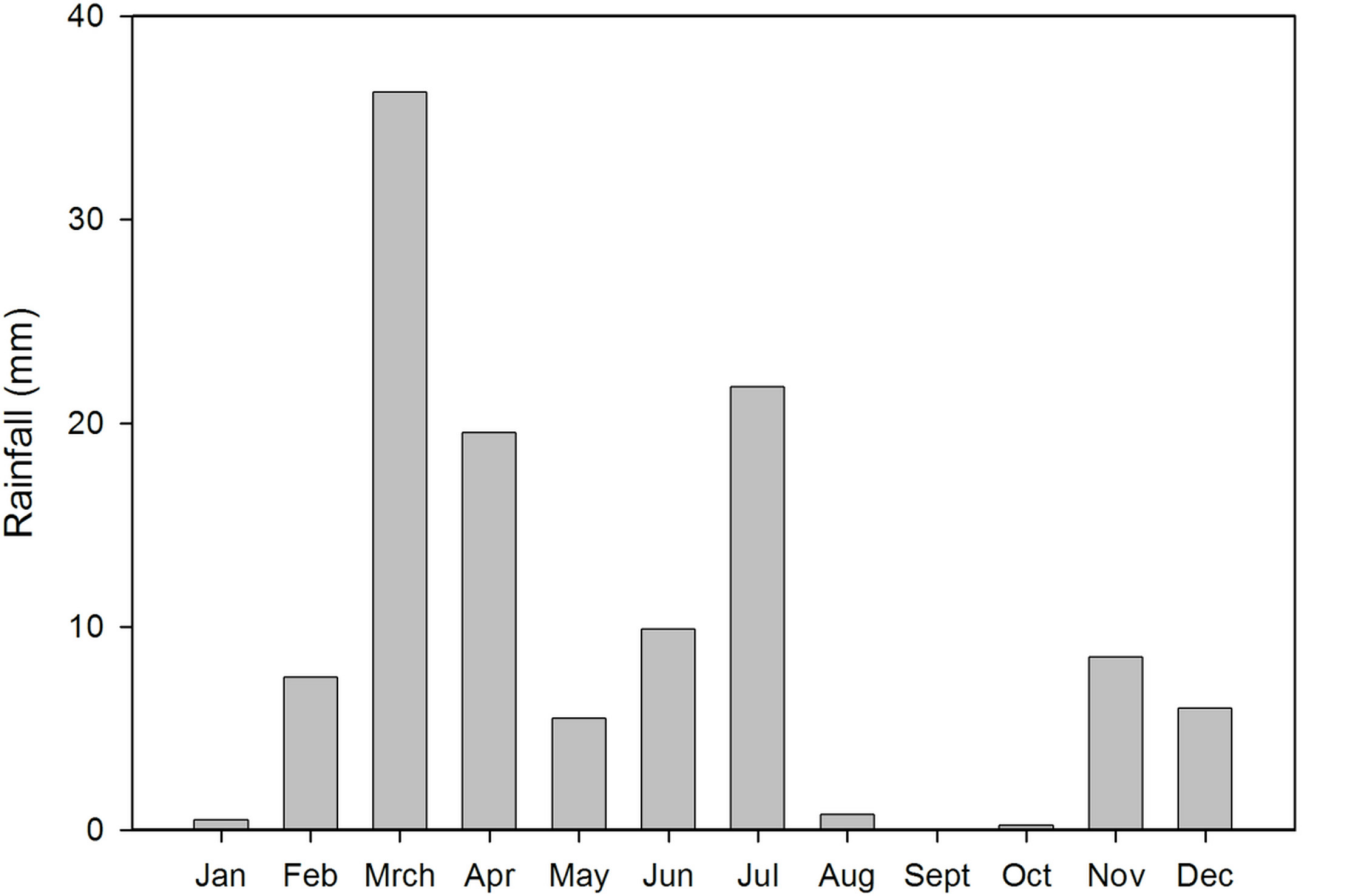
Total rainfall (mm) during the drought year of 2006. Conditions were particularly dry during our study period (28 Sept. to 26. Nov 2006).

We used a 700 x 1000 m site near Bundey Bore Station (33°54’16”S, 139°20’43”E) on unprotected privately owned land, in the mid-north of South Australia. The area is characterized by homogeneous chenopod shrubland, dominated by bluebush, *Maireana sedifolia*. It has a semi-arid climate with an annual median rainfall of 230mm (interquartile range [192, 280]; years 1925 to 2005). We conducted this study during the austral spring and early summer of 2006. The year of our study was a drought year with only 50 percent (116mm) of the median annual rainfall in the area, and it was the 6^th^ driest year since 1925 (Fig 1). During the two months preceding the start of our study there had been only 0.75mm of rain. During our study period (28 Sept to 26 Nov 2006) it rained at Bundey Bore station, 3 km from the study site, on only 6 days with a total of 8.25mm. Fig 2 shows the monthly rainfall pattern over the course of the year, illustrating the dry conditions during the period of our study.

### Data recording

During early spring (Aug-Sept) 2006 we captured 23 adult sleepy lizards in our study site (13 males, 10 females), that were part of a larger population occupying similar habitat surrounding the site. Lizards were measured (snout-to-vent length, SVL) and weighed at their first capture. We observed the spatial behaviour, activity and body condition of those lizards over 60 days in the spring and early summer (28 Sept to 26 Nov 2006), the period that contains most of their annual activity [18]. For this study, we divided the observations into an early period (the first 30 days), and a late period (the last 30 days).

Applying previously described methods [21], we attached data loggers to the dorsal surface of the tail of each lizard using surgical tape. The data loggers included a Global Positioning System (GPS) and a step counter and weighed 37g, or 4.9% of an average 750g adult lizard, and 7.7% of the lightest lizard in our study population. On every day of the 60 day study period, the units recorded the number of steps taken by each lizard every 2 min, and the GPS location every 10 min when lizards had taken at least one step in the previous ten minutes. Units sometimes failed or ran out of battery when lizards sheltered in burrows and could not be accessed for maintenance. Hence, the number of days when lizards were observed could be below 30 for each period (early period: median = 28.0, interquartile range [22.5, 28.0]; late period: median = 28.0, interquartile range [24.0, 28.0]). Furthermore, not all lizards were initially caught at the same time, and two lizards were recorded only during the late period. Units also included a radio transmitter, with unique frequency, allowing us to identify, locate and hand-capture each lizard, every 12 days to download data and renew batteries. Handling time of 15–30 min was excluded from the dataset. We have previously used data generated from these units to describe patterns of movement and social association in sleepy lizards [21–27]. We found no evidence that GPS loggers adversely affected lizard behaviour. Lizards foraged normally and we did not observe any behavioural lethargy when lizards were relocated and caught. At the end of the study, we removed the units and released all lizards. We detected no adverse effect of the units on the lizards. Sleepy lizards naturally shed their skin in the following months, which would remove any undetected damage to their skin.

### Identifying access to the dam

The study site contained an almost circular man-made dam, with an approximate radius of 30m, which held water during the study period. The dam was constructed to provide water for sheep that grazed in the area. It was the only source of free standing water in the study site, and annual plants remained growing in the moist soil around the edge of the dam until the end of the study, while those over the rest of the study site dried out earlier. We determined the coordinates of the centre of the dam with Google Earth, and confirmed the location of the dam with a handheld GPS device. We calculated the dam area as a circle with 30m radius around the centre location, which included the water body and the expansive dam walls. We then identified lizards that had access and contacted the dam if their GPS locations were recorded within the circle that we had specified as the dam area. We compared the behaviour and body condition of these lizards to those that did not have access to the dam. We further quantified the proportion of time (i.e. proportion of location records) each lizard spent at the dam, and investigated whether lizards spent more time at the dam during the late study period.

### Description of lizard spatial behaviour and body condition

Dry conditions were clearly visible throughout the area when the study started. Nevertheless, we assumed that the conditions would worsen and hypothesised a greater influence of the dam on lizard behaviour during the latter part of the season. We predicted an interaction effect between study period and access to dam on the lizard spatial behaviour. For each of the two 30 day periods we calculated home range size for each lizard as the 95% minimum convex polygon (MCP), using Ranges 8 [28]. Sleepy lizards are not active every day [29] and we defined periods of substantial lizard activity as the proportion of recording days when a lizard took more than 100 steps. We calculated lizard daily movement as the median number of steps taken per day, on days when at least one step was taken. Finally, we determined body mass for each lizard 1–3 times during each of the 30 day periods, and calculated each lizard’s median mass for each period. Then, for both periods together, we regressed the natural logarithm transformed values of median body mass against SVL for all lizards and used the unstandardized residuals as an index of individual lizard body condition [30].

### Analysis

All analyses were performed in IBM SPSS 20. We used the mixed function in SPSS to construct repeated measures linear models, which allowed us to include two lizards (one with and one without access to the dam), which were only observed in the second study period. We analysed the four dependent variables separately and square root transformed home range size and daily movement to achieve a normal distribution of the error terms of the models. In each model we included the explanatory factors *Access to dam* (yes/no) and *Study period* (early/late) as well as their interaction. A significant effect of *Access to dam* as well as of the interaction term *Access to dam x Study period*, would support our hypothesis of a behavioural difference between lizards that did or did not have access to the dam, as well as a stronger effect later in the season. Our models used a general covariance matrix with no relationships within the matrix. SPSS refers to this covariance structure as ‘unstructured’. We followed West et al. (2007) to select the model with the most suitable covariance structure, and determined that the models with the unstructured covariance matrix had greater explanatory power than the same models with the default covariance structure for repeated effects; that is a diagonal structure and heterogeneous variance. We compared the models with different covariance structures using restricted likelihood ratio tests, with p-values calculated using χ2 distributions (West et al. 2007). We did not exclude the interaction term from the model if it was not significant, because we were interested in the interaction effect. The data are available as supplementary information (S1 Dataset).

### Ethics statement

All procedures were formally approved by the Flinders University Animal Welfare Committee (AWC reference number E232) in compliance with the Australian Code of Practice for the Use of Animals for Scientific Purposes and we conducted our work under a Permit to Undertake Scientific Research (no A23436 15) from the South Australian Department of Environment, Water and Natural Resources.

## Results

**Fig 3:**
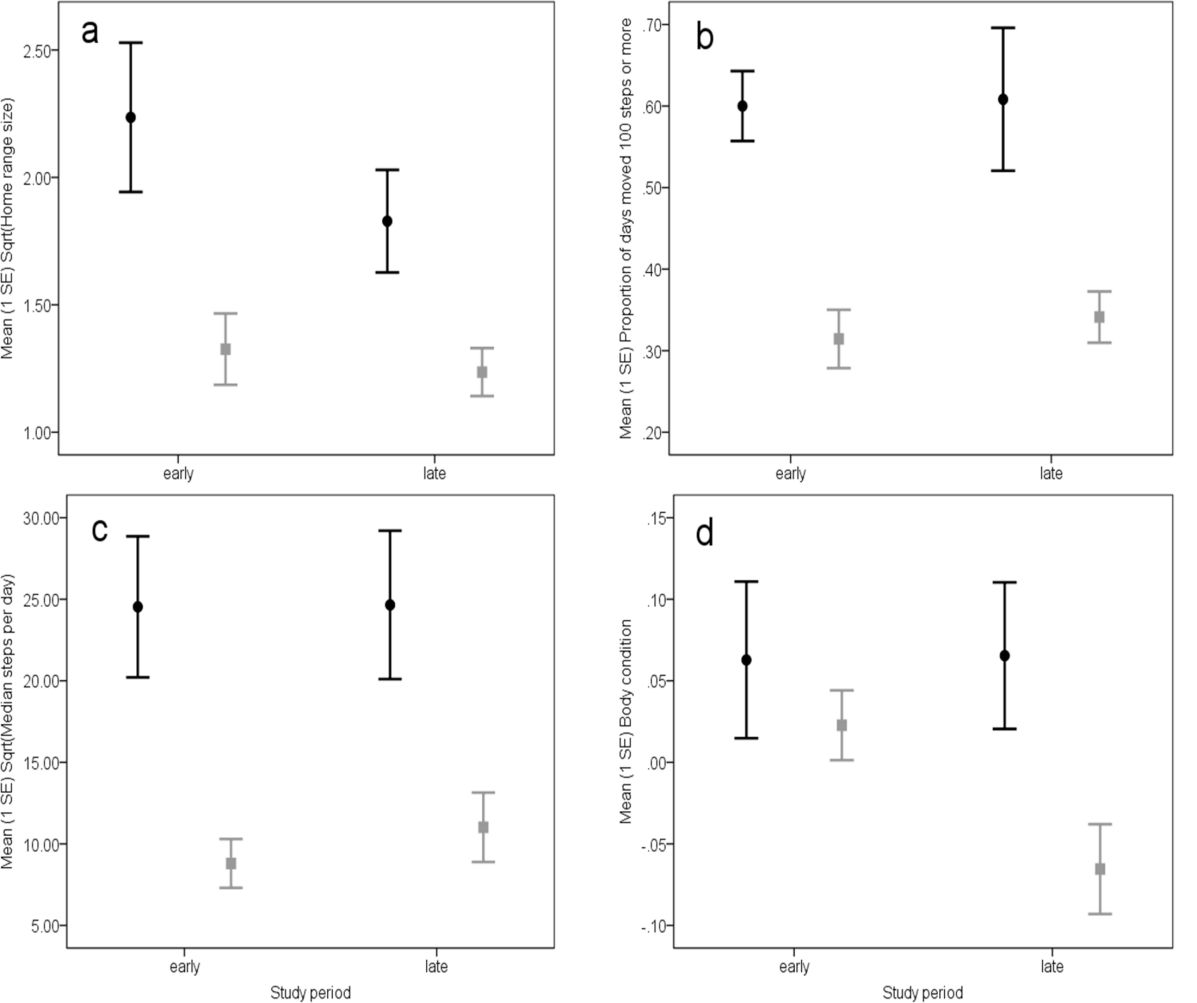
Effect of access to the artificial water point on lizard spatial behaviour and body condition during the early and late part of the study period. a) home range size, b) activity, c) movement, d) body condition. Black circles show lizards with access to the dam, and grey squares lizards without access.

We observed 23 adult lizards; six had access to the dam and 17 did not have access. Over the 60 days, we recorded a total of 633171 steps, with a median count of 46479 steps per lizard (interquartile range [31831, 62019]) for lizards with access to the dam and a median of 18482 steps per lizard (interquartile range [12544, 28736]) for lizards without access. We found that lizards with access to the dam had larger home ranges (Fig 3A), were substantially active on more days (Fig 3B) and moved more steps per day (Fig 3C) compared to lizards that did not have access to the dam (Table 1). None of the measures differed between early and late season, and we did not find any significant interactions *Access to dam x Study period* (Table 1). For those lizards with access to the dam, the proportion of time per lizard spent at the dam did not differ between the early season (median = 0.266, interquartile range [0.110, 0.500]) and late season (median = 0.338, interquartile range [0.031, 0.517]; Wilcoxon Signed Ranks Test *Z* = -0.674, *p* = 0.500). None of these results suggested any seasonal impact. However, the body condition showed a significant interaction effect of *Access to dam* x *Study period* (Table 1). While the two groups of lizards had similar body condition in the early period, they differed in the late period as those with dam access retained, but those without dam access lost body condition (Fig 3D).

**Table 1:** Lizard spatial behaviour and body condition.

	Home range size			Activity			Movement			Body condition		
	df	F	p	df	F	p	df	F	p	df	F	p
Intercept	20.397	245.018	**<0.001**	21.441	248.108	**<0.001**	20.131	104.594	**<0.001**	20.281	1.059	0.316
Access to dam	20.397	10.825	**0.004**	21.441	22.443	**<0.001**	20.131	18.633	**<0.001**	20.281	3.598	0.072
Study period	17.136	2.711	0.118	20.723	0.073	0.790	19.292	0.100	0.755	20.122	10.141	**0.005**
Access*Period	17.136	1.159	0.297	20.723	0.168	0.686	19.292	0.285	0.600	20.122	9.166	**0.007**

Results of the general linear model analyses in relation to whether lizards had access to the artificial watering point (dam) or not, and in relation to the early or late period of the study.

## Discussion

The year 2006 was unusually dry. In years with higher precipitation sleepy lizards changed their behaviour over the course of the active season, with lizards most active in spring, and becoming less active towards summer when ambient temperatures increase and annual plants dry out and become unavailable as food [18]. Lizards generally reduce activity in less suitable ambient conditions [8–10], consequentially resulting in smaller home ranges. During the early part of the current study, the home range size of lizards without access to the dam (median = 1.4 ha) was substantially smaller than the average home range size of about 4 ha previously reported for this species in locations close by [31, 32], whereas for those with access to the dam it was similar (median = 5.2). This suggests that in our current study, lizards away from the dam were already affected by the unusually dry ambient conditions. Over the course of the study, we found a consistent pattern that lizards with access to the dam showed different spatial behaviour, had larger home ranges, were substantially active on more days, and moved more per day than lizards with home ranges away from the dam. Lizards with access to the dam spent a similar proportion of their active time at the dam during both the early and the late period of our study. We acknowledge that our sample of six lizards with access to the dam was relatively small. Nevertheless, our combined results consistently support our interpretation that the dam and its surrounding area provided important resources, which lizards repeatedly visited over the whole study period.

In many animal species individuals move to areas with more abundant or higher quality resources when local resource levels decline. Spectacular examples are the mass migrations of ungulates [33, 34]. This poses two questions for our study. First, why did lizards with access to the dam maintain larger home ranges than lizards without access, instead of shifting their entire movement activity towards the dam and reducing home range size? One possible explanation is that the area around the dam lacked other important resources, for example shelter sites. Sleepy lizards establish a small non-random set of refuges which are repeatedly used and which are located within the inner exclusive core area of their normal home range [35, 36]. Home ranges are stable over time [32], and lizards in our study may have continued to use those well-established refuges located some distance from the dam. In addition, social organisation and social networks of the sleepy lizard are characterised by avoidance of some conspecific neighbours [21, 37]. Incidents of highly aggressive behaviour have been observed, or have been inferred from fresh wounds on the heads [38, 39]. Disruption of the social system risks increasing the frequency of these incidents. We suggest that in dry times, lizards traded-off the benefit of using the dam with the costs of shifting the home range, and disrupting the spatial and social organisation of the population.

Another important influence on home range size may be the increased grazing and trampling impact of livestock. This impact is often greatest around dams or other artificial watering points and decreases with distance, so that, while water is present, food plant availability sometimes may be reduced close to dams [3]. However, sheep stocking density in the study area was low. Nevertheless, after drinking at the dam, lizards may have moved to other areas, away from the dam, to avoid interactions with sheep and to find remaining plants. Although drier food plants may have been inadequate to sustain activity of the water stressed lizards away from the dam, lizards with access to water may have been able to benefit from them. We did not quantify food availability or sheep grazing pressure across the study site. Hence, we could not determine the relative importance of refuge or food availability in determining the larger home ranges of lizards with access to the dam.

A possible alternative explanation for the observed behavioural differences between lizards with and without access to the dam could be that lizards at the dam were disturbed by sheep visiting the dam and moved more to avoid interactions with them. However, availability of possible refuges to avoid interactions is high in the area. For example, Kerr et al. [40] reported an average refuge density of 44 bushes per 100 m^2^ in an area close to our study site. Furthermore, lizards with access to the dam were substantially active on more days than lizards without access, which contradicts the interpretation of greater avoidance behaviour, and lizards with access to the dam retained body condition, rather than losing it as would be predicted if they were stressed by sheep [41]. Taken together there is little support for the notion that sheep avoidance could influence the observed lizard spatial behaviour and activity patterns. However, empirical observations on how lizards respond to the presence of sheep would be needed to better understand the interspecies interaction.

The second question is why lizards away from the dam did not move towards the dam? One explanation is that those lizards were not aware of the dam. Some animal species have areas, for example breeding and communal shelter sites, where exchange of public information can occur, for instance about feeding opportunities [42]. There is currently no evidence that this communication operates in sleepy lizard populations, suggesting they may have limited social information regarding resource availability. The alternative, of lizards with a resource shortage exploring more widely the area around their usual home range and searching for better resources, may be costly and risky. Metabolic travel costs scale with the distance moved, and the travel costs for lizards, exploring with uncertain rewards may be too high relative to the unknown benefit of accessing resources at the dam, particularly if other important resources such as shelter or food are not available at the dam. Furthermore, a lizard that moves out of its own home range and crosses neighbouring ranges may incur additional social costs. Each summer these lizards reduce their activity as resources decline, and increased inactivity may be a safer response to drying conditions than searching for resources that may not be available. The stable spatial [32] and social [25] organisation of this lizard may lead to water deprived lizards staying in their established home ranges and reducing activity instead of moving around to find better resources.

Another important result of our study was that body condition was differentially affected over the study period depending on whether lizards did or did not have access to the dam. Lizards are ectotherms that behaviourally thermoregulate by basking and sheltering to compensate for environmental temperature variation [43]. Lizard body temperature affects basal metabolic rate and lizards can reduce energy expenditure when resources are scarce, both by decreasing activity [44], and by maintaining cooler body temperatures through changes in thermoregulatory behaviour [45]. Our findings are consistent with this behavioural strategy. Lizards away from the dam were substantially active on fewer days and moved less per day, resulting in smaller home ranges. Although these lizards had body conditions similar to lizards around the dam during the earlier period of the study, by the late period those lizards had significantly lower body condition than lizards around the dam. This suggests that, while reducing activity is a suitable short term strategy, if harsh conditions persist, then access to dams or other artificial watering points is of significant benefit and supports sustained lizard activity and movement, likely spent foraging, allowing those lizards to maintain their body condition. A broader interpretation is that access to some form of water has a major role in determining whether sleepy lizards will remain active. It explains the widely reported result that this species focuses its activity in the spring time and is largely inactive during similar temperature conditions later in the summer, as the landscape gets drier.

Lizards usually respond to unsuitable thermal, hydric or resource conditions with periods of extensive inactivity [18, 44]. Our findings suggest that local heterogeneity in access to an artificial water point resulted in spatially dependent behavioural variation among sleepy lizard individuals. This suggests that sleepy lizards have flexible responses to changing climatic conditions, depending on the availability of water. With a predicted increase in the frequency of droughts across southern Australia [46, 47], artificial watering points, such as the dams constructed by pastoralists to catch the run-off from infrequent rainfall events, may provide local favourable refugia for sleepy lizards and other species. This would be particularly important for species that are unable to regulate their water flux through food and cell respiration alone. However, altered spatial organisation and local aggregation around dams may have trickle-down effects and influence, predator-prey relationships, parasite transmission, mating systems and the strength of sexual selection. This warrants further investigations, highlighting that species apparently not under immediate threat from changed climate conditions, may still adjust their spatial ecology and behaviour in response to those climate changes.

## Supporting Information

S1 Dataset Dataset for analysis. (XLSX)
